# Gallstone Ileus of the Colon: Leave No Stone Unturned

**DOI:** 10.1155/2013/359871

**Published:** 2013-07-22

**Authors:** P. B. Salemans, G. F. Vles, S. Fransen, R. Vliegen, M. N. Sosef

**Affiliations:** ^1^Atrium Medical Center, Department of General Surgery, Henri Dunantstraat 5 6419 PC Heerlen, The Netherlands; ^2^Atrium Medical Center, Department of Radiology, Henri Dunantstraat 5 6419 PC Heerlen, The Netherlands

## Abstract

A case of gallstone ileus of the colon with illustrative pictures is presented, making the physicians more aware of this rare entity. Furthermore, the use of imaging modalities for diagnosis and decision making in management strategy is discussed.

## 1. Introduction

In 1941 L.G. Rigler described his famous triad of roentgen manifestations indicating gallstone ileus (GSI), that is, the association of an ectopic gallstone, bowel distension, and pneumobilia [1]. Rigler's triad, not to be confused with Rigler's sign, is present on a plain abdominal radiograph in merely 15% of GSI [2]. However, it is seen in over 77% on abdominal computed tomography (CT) scans [2]. The pathogenesis holds that due to episodes of calcifying cholecystitis a fistula develops between the gallbladder and the bowel, most often the duodenum [3]. A large gallstone is then able to migrate to the gastrointestinal tract causing mechanical ileus when (a) it is larger than 2.5 centimetres with concurrent bowel pathology, for example, tumor and diverticulitis [4–6], (b) it is larger than 5 centimetres, (c) several small calculi form an inspissated mass [7], or (d) there is faecal deposition on a small gallstone [8]. The most common locations of impaction are the terminal ileum and the ileocecal valve because of the anatomical small diameter and less active peristalsis [9].

GSI is a rare disease only accounting for 1–4% of all cases of mechanical intestinal obstruction [3]. Its incidence increases up to 25% in older females with extensive comorbidities [9]. Colonic obstruction due to gallstones is even more rare and accounts for 2–8% of all cases of GSI [10, 11]. Usually a gallstone enters the colon directly via a cholecystocolic fistula, but GSI of the colon has been reported with cholecystoenteric fistulas, indicating that the gallstone has somehow made it beyond the ileocecal valves [5]. It is a disease of high morbidity and mortality due to late presentation, advanced patient age, comorbid states, and most importantly the diagnostic challenge [12]. Since the 3 horsemen of colonic obstruction are malignancy, volvulus, and diverticular disease, the diagnosis of GSI of the colon is not usually considered. In about half of the cases the diagnosis is only made during laparotomy [12].

A case of GSI of the colon with illustrative pictures is presented, making the physician more aware of this rare entity. Furthermore, the use of imaging modalities for diagnosis and decision making in management strategy is discussed.

## 2. Case Report

A 78-year-old woman with a medical history of appendectomy, transient ischemic attack, cognitive dysfunction, type II diabetes mellitus, urinary and faecal incontinence, obesity, and hypercholesterolemia was admitted to the emergency department of our hospital because of abdominal pain, nausea, and vomiting. She had been constipated for three days. Physical examination revealed a mechanical ileus and a palpable mass in the left hemiabdomen. Laboratory studies were as follows: white blood count (WBC): 20.5 10∗9/L; C-reactive protein (CRP): 176 mg/L; bilirubin: 21.6 *μ*mol/L; gamma-glutamyl transpeptidase (*ϒ*GT): 67 U/L; alkaline phosphatase (AP): 97 U/L.

**Figure 1:**
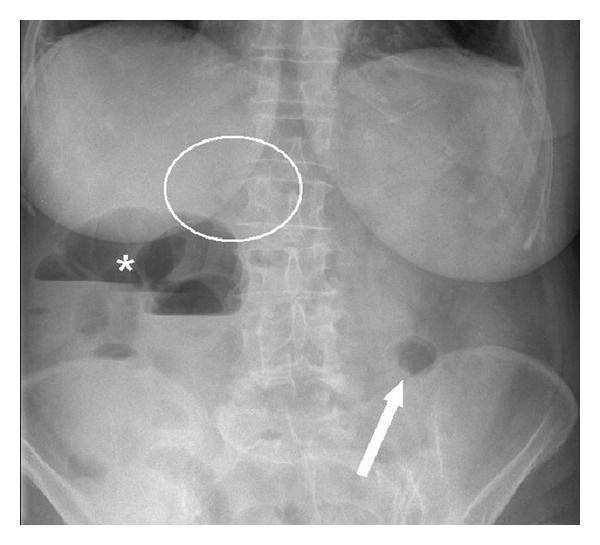
Plain abdominal radiograph showing Rigler's triad (pneumobilia indicated by the circle, ectopic gallstone indicated by the arrow, and bowel distension indicated by the asterisk).

**Figure 2:**
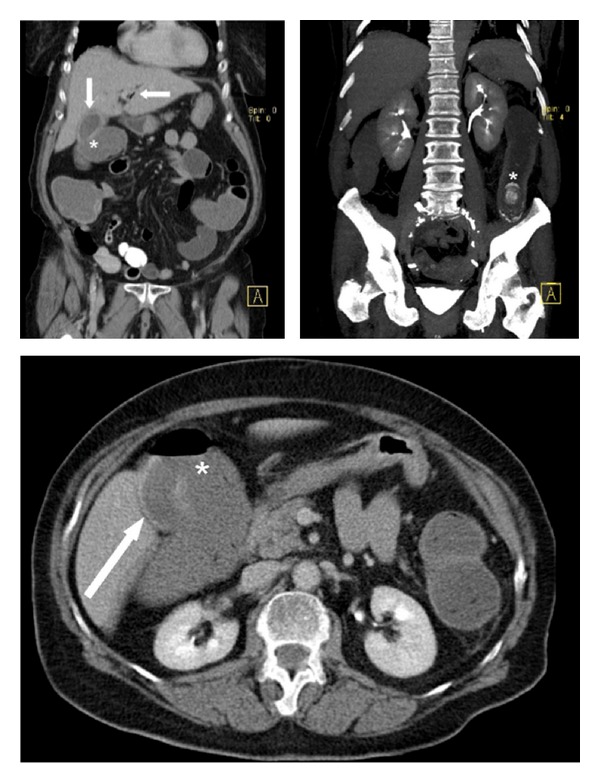
Abdominal CT scan showing a 6-centimetre radiopaque lesion in the descending colon, gall bladder wall thickening (arrow), a cholecystocolonic fistula (asterisk), pneumobilia (arrow), and an ectopic stone in the descending colon (asterisk).

A plain abdominal radiograph (Figure 1) showed a classical Rigler's triad. An abdominal CT scan with intraluminal contrast (Figure 2) furthermore demonstrated a cholecystocolic fistula, gallbladder wall thickening, and a six centimetre radiopaque lesion in the descending colon with induration of the adjacent mesocolic fat.

A conservative management strategy including nasogastric tube, intravenous antibiotics, and colon lavage was chosen because of the extensive comorbidities of the patient and because the gallstone was already in the descending colon. Spontaneous passage of gallstones was awaited as this has been reported in the literature [11, 13, 14]. The patient's clinical condition improved, and according to reports by the nursing staff the stone had passed with defecation.

**Figure 3:**
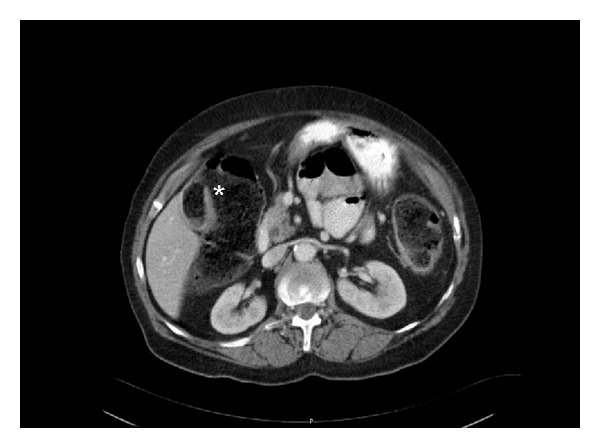
Follw-up abdominal CT scan showing a cholecystocolonic fistula with faeces in the gallbladder (asterisk).

**Figure 4:**
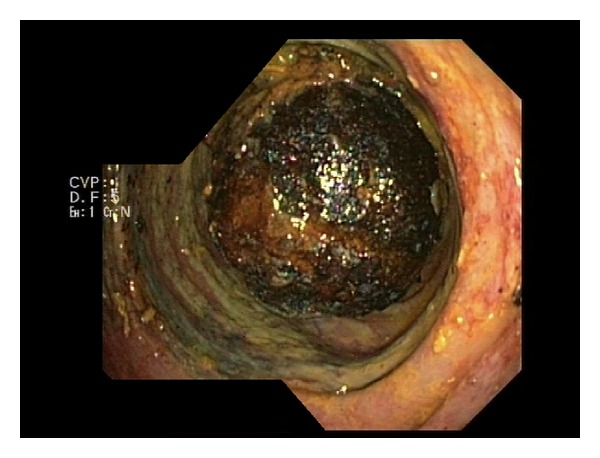
Endoscopic view of an obstructing and inextractable gallstone in the sigmoid colon.

However, seven days after discharge the patient was readmitted experiencing the same complaints as during the first admission. Laboratory studies had worsened: WBC: 27.2 10∗9/L; CRP: 338 mg/L; bilirubin: 17.3 *μ*mol/L; *ϒ*GT: 86 U/L; AP: 190 U/L. A new abdominal CT scan showed that the gallstone was still in situ but had passed onto the sigmoid colon and that the gallbladder was now containing faecal material (Figure 3). A colonoscopy revealed an obstructing gallstone at 20 centimetres from the anus causing cyanosis and edema of the adjacent colonmucosa (Figure 4). Attempts to extract the stone failed.

**Figure 5:**
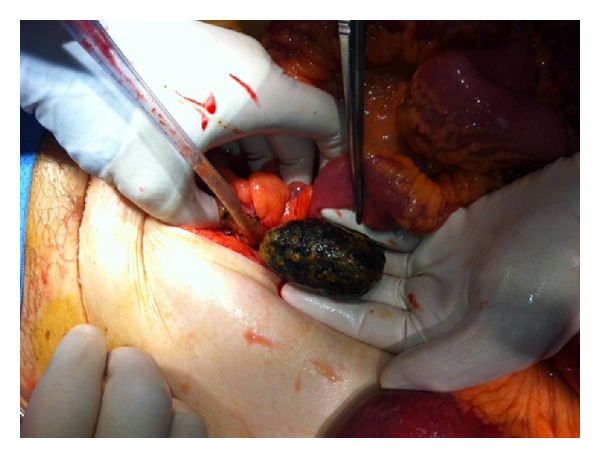
Surgical removal of the obstructing gallstone.

**Figure 6:**
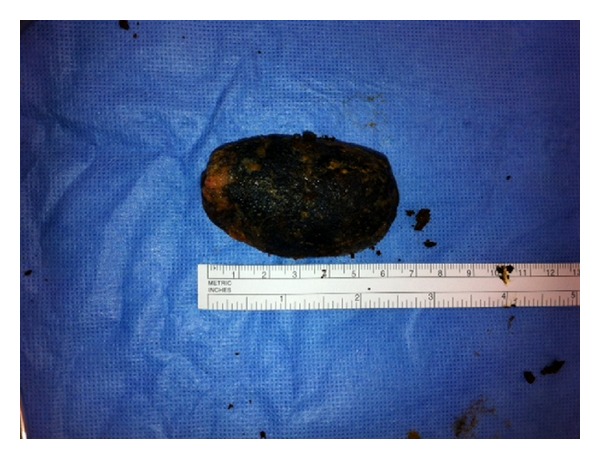
Removed gallstone measuring more than 6 centimetres.

In the subsequent hours the clinical situation of the patient deteriorated. An exploratory laparotomy showed a diffuse purulent peritonitis, dilatation of bowel, and a concrement in an ischemic sigmoid colon. It was decided to perform a cholecystectomy, a cholecystocolonic fistulectomy, and a sigmoidostomy to extract the stone (Figures 5 and 6) and to establish a double-barrel transverse colostomy. Relaparotomy because of clinical deterioration the next day demonstrated a perforated necrotic descending colon and fecal peritonitis. A subtotal colectomy with an ascending colostomy was performed. Unfortunately the patient passed away on the next day in the intensive care unit. Permission for autopsy was not obtained.

## 3. Discussion

GSI is a rare disease not usually considered by the physician. However, delayed or missed diagnosis may have severe consequences.

Plain abdominal radiographs have for long been the fundamental method to recognize the pathology. The main signs to acknowledge are Rigler's triad and Balthazar's sign, that is, air in the gallbladder [1, 15]. However, as previously described, in merely 15% of the cases the diagnosis can be made on plain abdominal radiographs  [2]. Therefore a high index of suspicion is crucial. An abdominal CT scan is considered the gold standard with a sensitivity of 93% and specificity of 100% [16]. It allows for accurately investigating the fistula between the gallbladder and the bowel and determining the degree of obstruction and the condition of the adjacent bowel mucosa. More sophisticated methods to identify the fistula between the biliary tract and the intestines are magnetic resonance cholangiopancreatography (MRCP) and drip infusion cholangiography CT (DIC-CT) [17]. Abdominal ultrasound can be used to confirm the presence of cholelithiasis and may also identify fistula, if present [18].

Besides diagnosis and decision making in management strategy, CT scan has a role in the followup of conservative treatment of GSI of the colon. With regard to the case presented, it was assumed that the gallstone had passed causing essential delay in surgery. Therefore we suggest that if a conservative management strategy is chosen, passing of the gallstone should be proven by CT scan.

Surgical relief of intestinal obstruction remains the mainstay of treatment. Recently, laparoscopy-guided enterolithotomy has become the preferred surgical approach in treating GSI [19]. Debate continues as to whether patients with GSI should have a combination procedure of enterolithotomy, cholecystectomy, and fistulectomy or enterolithotomy alone just to resolve the immediate obstruction [13]. Additionally, the nonsurgical treatment of GSI has been suggested, including endoscopic removal and shockwave lithotripsy, but this depends on the location of obstruction [14, 20].

In conclusion, a case of GSI of the colon with illustrative pictures is presented, hopefully making the physicians more aware of this rare entity. Our case illustrates that abdominal CT is the most appropriate noninvasive technique for diagnosis, treatment planning, and evaluation of success of conservative treatment.
